# Retrospective Cohort Analysis of TyG, TyG-SI, and TyG-Lac Indices as Predictors of 360-Day Mortality in Critically Ill Ischemic Stroke Patients

**DOI:** 10.3390/jcm15072680

**Published:** 2026-04-01

**Authors:** Chao Zhang, Weikan Wang, Huaibin Liang, Hao Fan, Jian-Ren Liu

**Affiliations:** Department of Neurology, Shanghai Ninth People’s Hospital, School of Medicine, Shanghai Jiao Tong University, Shanghai 200011, China; 1607376163@sjtu.edu.cn (C.Z.); 125056@sh9hospital.org.cn (W.W.); lianghuaibin@alumni.sjtu.edu.cn (H.L.);

**Keywords:** ischemic stroke, triglycerides, critical illness, insulin resistance, mortality

## Abstract

**Background**: This study aimed to compare the prognostic value of three surrogate insulin resistance (IR) markers for predicting 360-day mortality in critically ill patients with ischemic stroke (IS): the triglyceride–glucose (TyG) index, TyG–shock index (TyG-SI), and TyG–lactate (TyG-Lac). **Methods**: The study population comprised critically ill IS patients identified from the Medical Information Mart for Intensive Care (MIMIC) IV database. The main outcome was 360-day mortality. We employed multiple analytical approaches to examine relationships between the three biomarkers and mortality outcomes, including multivariable Cox proportional hazards models (Cox models), Kaplan–Meier survival analysis, and restricted cubic spline (RCS). Furthermore, receiver operating characteristic (ROC) curve analyses were conducted to assess the predictive capacity of these three indices. We performed ROC analyses to evaluate whether the IR index improved the discriminatory ability of a base model that included baseline variables significantly different between survivors and non-survivors. **Results**: Altogether, 812 patients with IS were included in the analysis. In Cox proportional hazards models, the TyG index was independently associated with higher 360-day mortality (HR, 1.68; 95% CI, 1.52–1.76). Similarly, both TyG-SI and TyG-Lac indices showed significant associations with 360-day mortality, with the HR (95% CI) of 1.24 (1.05–1.38) and 1.11 (1.08–1.23), respectively. Kaplan–Meier survival curves showed a progressive elevation in cumulative 360-day mortality across ascending quartiles of each index (TyG, TyG-SI, and TyG-Lac). ROC curve analysis revealed relatively better discriminatory ability of the TyG-SI compared to TyG and TyG-Lac for all-cause 360-day mortality prediction (area under the curve: 0.605 [0.578–0.623] vs. 0.566 [0.532–0.592] vs. 0.587 [0.532–0.614]). Furthermore, incorporation of either the TyG-SI or TyG index modestly improved the 360-day mortality prognostic accuracy of the base model (area under the curve [AUC], 0.701 for the base model vs. 0.723 for the base model + TyG-SI vs. 0.716 for base model + TyG index). **Conclusions**: When analyzed as continuous variables, each of the three indices demonstrated significant associations with 360-day mortality risk of critically ill IS populations. Moreover, both TyG-SI and TyG can improve the 360-day mortality predictive accuracy of the base model. Among the three indices, TyG-SI showed comparatively better discriminatory performance; however, the magnitude of AUC improvement was modest, and its clinical utility should be interpreted cautiously pending external validation.

## 1. Introduction

Ischemic stroke (IS) remains among the most significant contributors to death and functional impairment globally [1,2]. Although endovascular treatment and intravenous tissue plasminogen activator (t-PA) have been established, considerable risk for unfavorable clinical prognosis in IS patients remains substantial, especially among critically ill individuals [3,4,5]. Accumulating evidence has identified triglycerides (TG) and diabetes as major risk factors for cerebrovascular disease [6,7]. Insulin may exert neuroprotective effects by attenuating ischemic injury and oxidative stress, limiting tissue damage after cerebral ischemia, and regulating cholesterol metabolism in neurons and astrocytes [8,9]. Insulin resistance (IR) is a critical component of metabolic syndrome and is defined by decreased responsiveness of target tissues or organs to insulin [10]. Multiple investigations have established robust associations between insulin resistance and IS development [11,12,13]. Insulin resistance may weaken insulin’s neuroprotective effects, including autophagy inhibition and oxidative stress reduction, thus worsening cerebral injury [14,15]. IR is closely linked to glucose dysregulation, lipid accumulation, and obesity [16]. Therefore, non-insulin-based surrogate markers have been utilized in epidemiological studies to evaluate IR. Calculated from triglyceride and glucose levels, the triglyceride–glucose (TyG) index serves as a convenient IR biomarker, providing superior cost-effectiveness and time-efficiency versus traditional methodologies like hyperinsulinemic-euglycemic clamp [17]. Previous research has demonstrated the independent prognostic value of the TyG index for predicting stroke recurrence, incidence, and mortality in patients with IS [18,19]. The shock index (SI) primarily reflects tissue perfusion status and is closely associated with massive transfusion requirements, intensive care unit (ICU) stay duration, and death rates [20,21]. Lactate, similar to glucose, can also serve as a metabolic substrate [22]. However, excessively elevated lactate levels often indicate tissue hypoxia. Bruno Levy et al. revealed significant associations between lactate levels during ICU admission and mortality outcomes (*p* < 0.0001) [23]. To improve the prognostic utility of the TyG index in the ICU setting, we constructed two composite markers, TyG-SI and TyG-Lac, and compared their performance in predicting short- and long-term mortality in patients with IS, with the aim of providing more informative prognostic tools for clinicians.

## 2. Method

### 2.1. Study Participants

This retrospective analysis examined health-related information derived from the MIMIC-IV database (version 3.0). The database contains clinical data of patients admitted to the intensive care unit at Beth Israel Deaconess Medical Center (BIDMC) spanning 2008 through 2022. Database access and data extraction were performed by one investigator (ZC; Certification Number 73921362). Patients with ischemic stroke were identified and enrolled based on ICD-9 and ICD-10 codes.

Patients were eligible for inclusion if they met all of the following criteria:

(1) Age ≥ 18 years;
(2) A diagnosis of ischemic stroke identified by ICD-9/ICD-10 codes;
(3) Admission to the ICU;
(4) ICU length of stay ≥ 24 h;
(5) Availability of the essential clinical and laboratory variables required for calculation of TyG-related indices within the first 24 h after ICU admission, including triglycerides, glucose, lactate, heart rate (HR), and systolic blood pressure (SBP).

Exclusion criteria included the following:

(1) Missing data on essential variables required for index construction; or
(2) In cases of multiple ICU admissions, only the first eligible admission meeting the study criteria was analyzed, whereas all subsequent ICU admissions were excluded to avoid within-patient duplication and preserve the independence of observations.

To further ensure methodological transparency, repeated records within the same ICU admission were handled as follows: when multiple measurements of the same variable were available during the first 24 h after ICU admission, the earliest available value was used for baseline assessment and index calculation, so that all predictors consistently reflected the initial ICU status of each patient.

### 2.2. Parameter Extraction

PostgreSQL software (v13.7.2) and Navicat Premium (version 16) were utilized for data extraction. Baseline variables were selected based on their potential influence on individual cardiovascular risk profiles. The extracted parameters were organized into three main categories: (1) demographic variables, including age, sex, weight, height, and body mass index (BMI); (2) comorbid conditions, encompassing heart failure, atrial fibrillation, diabetes mellitus, respiratory failure (RF), and acute kidney injury (AKI); (3) laboratory biomarkers, comprising hemoglobin, platelet, red blood cell (RBC), white blood cell (WBC), glucose, potassium, sodium, calcium, magnesium, prothrombin time (PT), partial thromboplastin time (PTT), creatinine, blood urea nitrogen (BUN), alanine aminotransferase (ALT), aspartate aminotransferase (AST), lactate, triglycerides, low-density lipoprotein cholesterol (LDL-C), and high-density lipoprotein cholesterol (HDL-C); (4) admission disease severity scores: the Sepsis-related Organ Failure Assessment score (SOFA), the acute physiology score III (APS III), the simplified acute physiology score II (SAPS II), Oxford Acute Severity of Illness Score (OASIS), and Glasgow coma scale (GCS)

For individuals with repeated measurements during the first 24 h after ICU admission, we used the first available value for demographic and hemodynamic variables and the earliest laboratory value within the initial 24 h window for index calculation whenever possible, in order to better reflect baseline status at ICU entry. Severity scores were extracted according to their corresponding database definitions within the same early ICU period.

The TyG index was determined using the following equation:

TyG = ln{[TG (mg/dL) × glucose (mg/dL)]/2}

Notably, glucose values represented the first available measurement within 24 h of ICU admission rather than strictly fasting glucose, which is an inherent limitation of database-derived TyG indices.

The Shock Index (SI) was computed as:

$$\mathrm{SI}=\frac{\mathrm{Heart}\;\mathrm{Rate}\;(\mathrm{beats}/\min)}{\mathrm{Systolic}\;\mathrm{Blood}\;\mathrm{Pressure}\;(\mathrm{mmHg})}$$

The TyG-SI composite index was defined as:

TyG-SI=TyG×SI

The TyG-Lac composite index was defined as:

TyG-Lac=TyG×Lactate (mmol/L)

All laboratory parameters and illness severity assessments were obtained from measurements recorded during the initial 24 h window following ICU admission.

### 2.3. Outcomes

The observation period began on the date of ICU admission. The 360-day mortality served as the primary endpoint, with 180-day mortality and in-hospital mortality designated as secondary outcomes.

### 2.4. Statistical Analysis

Continuous variables were presented as mean ± standard deviation or median with interquartile range (IQR), while categorical parameters were summarized as numbers and percentages. Chi-square test or Fisher’s exact test was utilized for categorical variable comparisons. For continuous variables, either Student’s *t*-test or the Wilcoxon rank-sum test was applied based on data distribution. Missing data were handled according to a prespecified protocol after assessing the proportion of missingness for all candidate variables (Supplementary Table S1). Variables required for the calculation of TyG, TyG-SI, and TyG-Lac were considered essential; therefore, patients with missing values for these variables were excluded during the screening stage. Among the remaining covariates, variables with >10% missingness were excluded from multivariable analyses, variables with 5–10% missingness were imputed using multiple imputation by chained equations (MICE; 10 imputations), and variables with <5% missingness were imputed using median values for continuous variables and mode values for categorical variables [24]. Continuous variables were additionally winsorized at the 1st and 99th percentiles to reduce the influence of extreme values. To evaluate the potential impact of excluding patients with incomplete essential laboratory data, we performed a sensitivity analysis comparing baseline characteristics between included and excluded patients; no significant differences were observed in age, sex, or major comorbidities (all *p* > 0.05), suggesting that this exclusion was unlikely to introduce substantial systematic bias. Nevertheless, a potential risk of selection bias cannot be completely excluded, as patients with missing laboratory data may represent a clinically distinct subgroup.

Abnormal values in variables were addressed through a winsorization method, with cutoff points set at the 1st and 99th percentiles. The associations of the TyG index, TyG-SI, and TyG-Lac with mortality risk were evaluated using Kaplan–Meier (K–M) survival curves combined with Cox models. Covariates for multivariable adjustment were selected a priori based on clinical relevance, prior literature, and data completeness. Baseline differences between survivors and non-survivors were examined descriptively and were used only as supportive information rather than the sole criterion for covariate inclusion. Because several severity scores included in the analysis (SOFA, APS III, SAPS II, and OASIS) reflect overlapping aspects of acute illness severity, we assessed potential multicollinearity prior to final model fitting using pairwise correlations and variance inflation factors (VIFs). The VIFs were 2.31 for SOFA, 3.42 for APS III, 4.15 for SAPS II, and 2.87 for OASIS, respectively. As all VIFs were below the conventional threshold of 5, these findings did not suggest problematic multicollinearity. Two multivariable models were then constructed to evaluate the associations between TyG-related indices and mortality outcomes. The initial model (Model 1) incorporated adjustments for patient age, atrial fibrillation, respiratory failure, and acute kidney injury. A more comprehensive second model (Model 2) extended these adjustments to include additional parameters: age, atrial fibrillation, respiratory failure (RF), acute kidney injury (AKI), SOFA, APS III, SAPS II, OASIS, hemoglobin, RBC, potassium, prothrombin time, and urea nitrogen. Restricted cubic spline (RCS) examined dose–response patterns between the three insulin resistance (IR) indices and mortality outcomes. ROC curve analysis compared predictive performance, sensitivity, and specificity across the three IR metrics. Two-tailed statistical significance was established at *p* < 0.05. Statistical analyses employed R software (version 4.4.3) and IBM SPSS Statistics (version 31.0, Armonk, NY, USA).

## 3. Results

### 3.1. Baseline Characteristics

**Figure 1:**
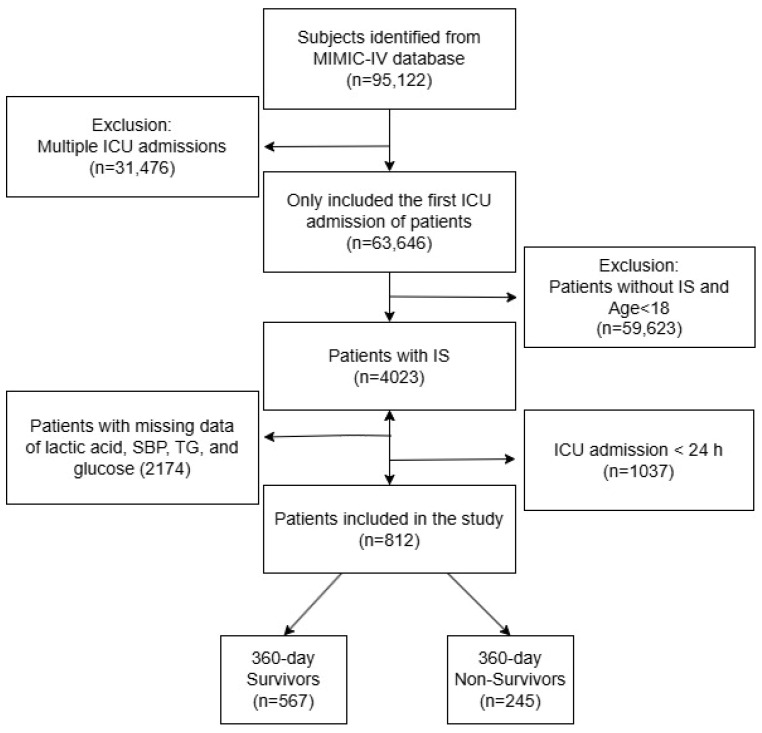
Flowchart of patient selection criteria in the study cohort.

Data screening identified 812 IS patients from the MIMIC-IV database who met enrollment criteria (Figure 1). As this was an exploratory study using existing clinical data, no a priori sample size calculation was performed. Sample size was determined by data availability. Patients were stratified by 360-day mortality into survivors (*n* = 567, 69.8%) and non-survivors (*n* = 245, 30.2%). Compared to survivors, non-survivors demonstrated significantly greater age and exhibited higher prevalence rates of AF, RF, and AKI. Additionally, non-survivors exhibited significantly elevated illness severity scores (SOFA, APS III, SAPS II, OASIS) and higher levels of potassium, urea nitrogen, and triglycerides compared to survivors (Table 1).

The proportion of missingness for candidate covariates is summarized in Supplementary Table S1. Variables necessary for the calculation of TyG-related indices were complete in the final study cohort by design after screening. For non-essential covariates, missing values were handled according to the prespecified imputation strategy described in Section 2.

A total of 95,122 subjects were initially identified from the MIMIC-IV database. After exclusion of repeated ICU admissions, patients without ischemic stroke (IS), patients aged < 18 years, patients with missing data for lactate, systolic blood pressure (SBP), triglycerides (TG), or glucose, and patients with an ICU stay of <24 h, 812 critically ill patients with IS were included in the final analysis. These patients were further classified as 360-day survivors (*n* = 567) and non-survivors (*n* = 245).

### 3.2. Associations of TyG Index and Mortality Risk

**Figure 2:**
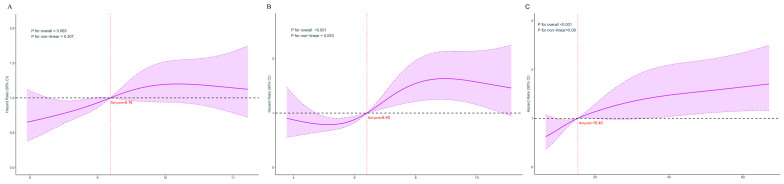
Restricted cubic spline curves showing associations of the three IR-related indices with 360-day mortality among patients with IS. (**A**) TyG index. (**B**) TyG-SI. (**C**) TyG-Lac. Solid lines represent adjusted hazard ratios (HRs), and shaded areas represent 95% confidence intervals (CIs). HRs were derived from multivariable Cox proportional hazards models. The solid purple line shows the estimated hazard ratio (HR), and the purple shaded region indicates the 95% confidence interval (95% CI). The black dashed horizontal line represents HR = 1.0, while the red vertical dotted line denotes the reference point.

**Figure 3:**
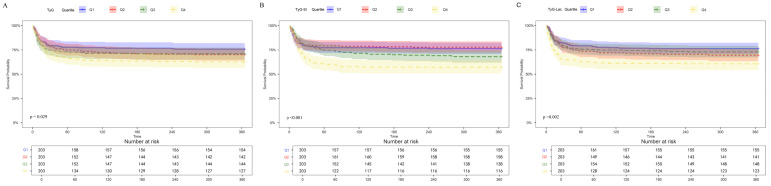
K–M survival analysis curves of 360-day mortality among patients with IS. (**A**) TyG index. (**B**) TyG-SI. (**C**) TyG-Lac. Differences between groups were compared using the log-rank test. TyG, triglyceride–glucose index; TyG-SI, triglyceride–glucose–shock index; TyG-Lac, triglyceride–glucose–lactate index.

Cox proportional hazards modeling demonstrated TyG index, when expressed as a continuous parameter, showed significant associations with 360-day mortality risk in both the unadjusted model (hazard ratio [HR] 1.68; 95% confidence interval [CI] 1.52–1.76) and the completely adjusted model (HR 1.32; 95% CI 1.05–1.43). Included patients were subsequently stratified into four equal groups according to quartile of TyG: Q1 (7.89–8.38), N = 203; Q2 (8.38–8.97), N = 203; Q3 (8.97–9.42), N = 203; and Q4 (9.42–11.21), N = 203. Compared with Q1, the highest quartile (Q4) demonstrated significant associations with 360-day mortality risk across all models: unadjusted (HR 1.88; 95% CI 1.28–2.76), Model 1 (HR 1.79; 95% CI 1.24–2.59), and Model 2 (HR 1.64; 95% CI 1.15–2.36) (Table 2). Moreover, the RCS model confirmed a linear dose-dependent relationship between TyG index elevation and 360-day mortality risk (*p* for non-linearity = 0.307; *p* for overall = 0.035) (Figure 2A). K–M curves showed that cumulative all-cause 360-day mortality increased progressively across TyG quartiles (Q1: 20.0%; Q2: 24.9%; Q3: 24.1%; Q4: 31.0%; *p* = 0.029) (Figure 3A).

### 3.3. Associations of TyG-SI and Mortality Risk

Cox regression demonstrated that TyG-SI, when modeled as a continuous parameter, exhibited significant associations with 360-day mortality, in an unadjusted model (HR 1.24; 95% CI 1.05–1.38) and a completely adjusted model (HR 1.11; 95% CI 1.04–1.19). For categorical analysis, TyG-SI was divided into four quartiles: Q1 (3.61–4.49), Q2 (4.49–5.87), Q3 (5.87–7.07), Q4 (7.07–11.12), it was also associated with an increased risk of all-cause mortality over 360 days in both the unadjusted model (Q1 vs. Q2: HR 0.92; 95% CI 0.61–1.38; Q3: HR 1.32; 95% CI 0.91–1.92; Q4: HR 1.96; 95% CI 1.38–2.79; trend *p* <0.001) and minimally adjusted Model 1 (Q1 vs. Q2: HR, 0.92; 95% CI 0.61–1.38; Q3: HR 1.39; 95% CI 0.96–2.03; Q4: HR 2.22; 95% CI 1.55–3.19; trend *p* <0.001) and demonstrated an ascending pattern corresponding to elevated TyG-SI values. Moreover, following adjustment for laboratory variables, IS patients in the higher TyG-SI quartiles exhibited progressively increased 360-day mortality incidence, compared with Q1, the HRs (95% CIs) were 0.85 (0.56–1.29) for Q2, 1.25 (0.85–1.84) for Q3, and 1.98 (1.37–2.88) for Q4, with a significant trend across quartiles (*p* for trend < 0.001) (Table 2). RCS confirmed 360-day mortality increased linearly as TyG-SI values rose (Figure 2B Nonlinear *p* = 0.053). K-M survival curves demonstrated that cumulative 360-day all-cause mortality escalated progressively across TyG-SI quartiles (Q1: 19.6%; Q2: 18.4%; Q3: 26.5%; Q4: 35.5%), with significant between-group differences (*p* < 0.001) (Figure 3B).

### 3.4. Association of TyG-Lac Index and Mortality Risk

TyG-Lac showed significant predictive associations with 360-day mortality across sequential models: unadjusted model (HR 1.11; 95% CI 1.08–1.23), minimally adjusted Model 1 (HR 1.24, 95% CI 1.07–1.32), and fully adjusted Model 2 (HR 1.18, 95% CI 1.01–1.21). IS patients were then classified into quartile groups according to the TyG-Lac index: Q1 (6.21–9.03), Q2 (9.03–13.36), Q3 (13.36–18.83), Q4 (18.83–68.01). Cox proportional risk analysis in completely adjusted model demonstrated that patients in the uppermost quartile (Q4) faced substantially greater 360-day mortality risk than those in the lowest quartile (Q1), compared with Q1, the HRs (95% CIs) were 1.30 (0.89–1.91) for Q2, 1.09 (0.73–1.63) for Q3, and 1.75 (1.20–2.52) for Q4, with a significant trend across quartiles (*p* for trend = 0.008). (Table 2). RCS analysis revealed that the TyG-Lac index was nonlinearly associated with the risk of 360-day mortality (Figure 2C, *p* for nonlinearity = 0.008). Kaplan–Meier survival analysis revealed significant differences in cumulative mortality across quartiles (Q1–Q4: 19.6%, 25.3%, 22.4%, 32.7%, *p* = 0.002) (Figure 3C).”

### 3.5. ROC-Based Evaluation of TyG, TyG-SI, and TyG-Lac

**Figure 4:**
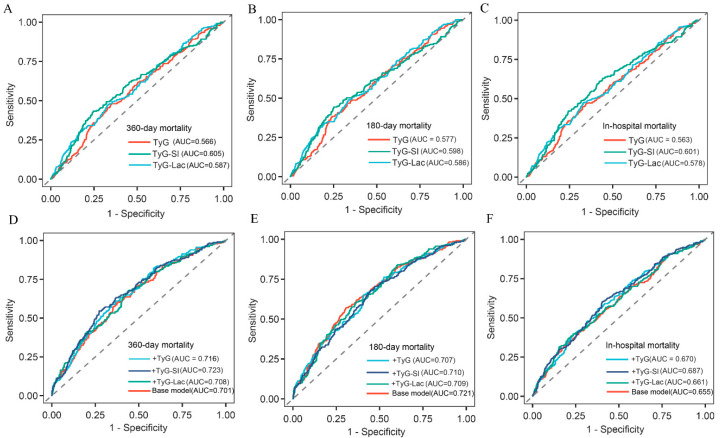
ROC curves for mortality prediction with IR indices. (**A**–**C**) Comparison of the predictive performance of TyG, TyG-SI, and TyG-Lac for 360-day mortality (**A**), 180-day mortality (**B**), and in-hospital mortality (**C**). (**D**–**F**) Comparison of the base risk model and the base model with the addition of TyG, TyG-SI, or TyG-Lac for predicting 360-day mortality (**D**), 180-day mortality (**E**), and in-hospital mortality (**F**). The area under the ROC curve (AUC) for each model is shown in the corresponding panel. TyG, triglyceride–glucose index; TyG-SI, triglyceride–glucose–shock index; TyG-Lac, triglyceride–glucose–lactate index.

**Figure 5:**
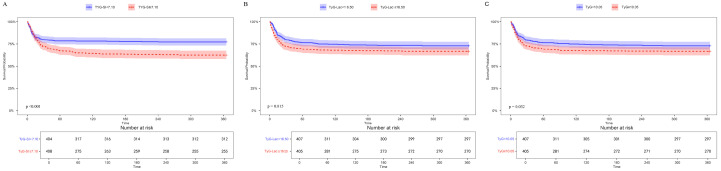
K–M survival analysis curves of 360-day mortality among patients with IS. (**A**) TyG-SI. (**B**) TyG-Lac. (**C**) TyG. Patients were categorized into two groups according to the corresponding cutoff value for each index. The blue solid line and red dashed line represent the low- and high-index groups, respectively, and the shaded areas indicate 95% confidence intervals. Differences between groups were compared using the log-rank test. TyG, triglyceride–glucose index; TyG-SI, triglyceride–glucose–shock index; TyG-Lac, triglyceride–glucose–lactate index.

ROC analysis evaluated the mortality prediction performance of three IR indices in IS patients (Figure 4). TyG-SI showed only a relatively higher discrimination than TyG-Lac and TyG, and the increase in AUC was limited, suggesting modest clinical improvement: 360-day mortality (AUC: 0.605 [95% CI 0.578–0.623] vs. 0.587 [0.532–0.614] vs. 0.566 [0.532–0.592]), 180-day mortality (AUC: 0.598 [0.576–0.631] vs. 0.577 [0.553–0.622] vs. 0.586 [0.567–0.627]), and in-hospital mortality (AUC: 0.601 [0.576–0.642] vs. 0.563 [0.545–0.602] vs. 0.578 [0.549–0.638]) (Table 3). The optimal cutoff values for predicting the primary outcome, 360-day mortality, were 7.10 for TyG-SI, 16.50 for TyG-Lac, and 10.05 for TyG. Based on these cutoff values, patients were stratified into high-parameter and low-parameter groups for each index (TyG-SI: ≥7.10 and <7.10; TyG-Lac: ≥16.50 and <16.50; TyG: ≥10.05 and <10.05). Both Cox proportional hazards modeling and K-M survival analysis demonstrated that the high-parameter groups had significantly greater mortality rates when compared with the low-parameter groups across all three indices (Figure 5).

Finally, we evaluated whether TyG-based IR indices improved upon a base model containing demographic, clinical, and laboratory variables (age, atrial fibrillation, respiratory failure, acute kidney injury, SOFA, APS III, SAPS II, OASIS, hemoglobin, RBC, potassium, prothrombin time, and urea nitrogen). Although TyG-SI and TyG-Lac each showed better predictive performance than the TyG index as standalone predictors across all mortality endpoints, adding these three indices individually to the base model resulted in heterogeneous and limited incremental improvements in predicting each mortality endpoint. TyG-SI and TyG yielded statistically significant improvements in discrimination for 360-day mortality when added to the base model, whereas TyG-SI and TyG-Lac showed statistically significant improvement for in-hospital mortality. However, the absolute increases in AUC were small, indicating that these indices may provide incremental rather than transformative prognostic information (Figure 4, Table 3). However, none of the three TyG-based IR indices provided significant incremental predictive value for 180-day mortality beyond the base model.

## 4. Discussion

We systematically evaluated the associations of three IR indices with clinical outcomes in critically ill patients with IS. This is the first study to assess the associations of TyG-SI and TyG-Lac with long-term mortality in this population. All three indices showed significant associations with 360-day mortality when analyzed as continuous variables. Addition of the TyG or TyG-SI improved the prediction of 360-day mortality, whereas TyG-SI and TyG-Lac improved the prediction of in-hospital mortality.

The TyG index, derived from triglycerides and fasting blood glucose, has emerged as a promising marker for metabolic disorders and cardiovascular disease [25,26,27]. Several clinical trials have explored correlations between the TyG index values and cardiovascular disease incidence and mortality across general populations. Cumulative TyG index measurements, capturing chronic insulin resistance burden, demonstrated positive correlations with ischemic stroke risk, whereby extended exposure duration corresponded to progressively elevated hazard ratios (HR = 1.30–1.26) [6]. Liu et al. evaluated the TyG index and its correlation with increased 28-day and 180-day mortality risk among critically ill patients under age 60 and in those with stroke [28]. A cohort analysis involving 7569 hypertensive participants from rural China, baseline TyG index demonstrated linear associations with cumulative ischemic stroke incidence, with stroke risk increasing proportionally to baseline TyG index elevation, particularly pronounced at TyG index ≥ 8.8 [29]. Their study evaluated the association between the TyG index and long-term outcomes in patients with hypertensive ischemic stroke. Unlike previous studies, we used a U.S. intensive care database to evaluate the prognostic value of the TyG index for both short-term and long-term outcomes in critically ill patients with acute ischemic stroke. In addition, we observed a strong association between the TyG index and 360-day mortality. Restricted cubic spline analysis showed that a higher TyG index, particularly at values ≥ 9.19, was associated with worse outcomes, possibly because hyperglycemia and dyslipidemia both contribute to increased mortality risk.

To our knowledge, this study is the first to introduce TyG-SI and TyG-Lac variables to investigate their relationship with long-term mortality outcomes in ICU-admitted IS patients. The SI, derived from heart rate and systolic blood pressure measurements, offers a simple yet effective prognostic marker for evaluating disease severity and patient prognosis in critical care environments. Studies have demonstrated that SI ≥ 1 can effectively predict early transfusion requirements in polytrauma patients, though its predictive capacity for length of in-hospital stay and mortality remains limited [20,30]. In stroke patients with concurrent circulatory failure (such as cardiogenic shock), the SI may be elevated, indicating circulatory decompensation and correlating with adverse outcomes. Moreover, severe shock may induce stress-induced hyperglycemia, thereby affecting the TyG index [31,32]. Similarly, elevated lactate levels in critically ill patients often indicate metabolic imbalance and serve as an important marker of tissue hypoxia [33,34,35]. Lactate accumulation and elevated TyG index both indicate dysregulation of glucose and lipid metabolism. Higher TyG index potentiates insulin resistance through aggravation of mitochondrial dysfunction, including enhanced lactate generation [36,37]. In our study, TyG, SI, and lactate were all independent predictors of IS. By combining the TyG index with SI and serum lactate to form composite indices, we further optimized risk assessment for stroke patients.

IR reduces NO bioavailability via endothelial nitric oxide synthase (eNOS) downregulation, promoting vasoconstriction, oxidative stress, and pro-inflammatory endothelial activation, impairing cerebral autoregulation and microvascular integrity [38].

Systemic inflammation: Elevated TG and glucose dysregulation activate NF-κB signaling, promoting IL-6, TNF-α, and CRP release, amplifying neuroinflammation and expanding the ischemic penumbra [39,40].

IR-associated mitochondrial dysfunction promotes anaerobic glycolysis and lactate accumulation, which is particularly detrimental in the energy-compromised post-ischemic brain [41,42].

IR-associated dyslipidemia and hyperglycemia promote microvascular rarefaction, pericyte dysfunction, and blood–brain barrier disruption, impairing cerebral perfusion reserve [12,43].

Plasma lactate reflects tissue hypoperfusion and hemodynamic compromise in acute critical illness [44]. Although Xiaoyi Xiong et al. have reported that elevated lactate can exacerbate ischemic brain injury by promoting protein lactylation (Kla), direct evidence that plasma lactate per se contributes to ischemic brain injury remains lacking [41]. In this context, the TyG-Lac composite may integrate chronic metabolic insulin resistance burden with acute circulatory stress and is biologically promising as a dual-pathway prognostic indicator for post-stroke injury.

Our findings are broadly consistent with prior investigations. Yang et al., in a systematic review and meta-analysis of 18 studies, confirmed that a higher TyG index was significantly associated with increased ischemic stroke risk and recurrence (pooled OR = 1.58, 95% CI: 1.19–1.89) [18]. Cai et al. specifically examined the TyG index in critically ill patients from MIMIC-IV and found significant associations with hospital mortality and ICU mortality in stroke subgroups [45], which aligns with our mortality findings. However, none of these prior studies evaluated composite indices incorporating hemodynamic (SI) or metabolic (lactate) parameters, which represents the primary novelty of the present work. Our demonstration that TyG-SI outperforms TyG alone suggests that integrating circulatory status into IR assessment provides incremental prognostic information in the ICU setting.

Moreover, we compared the prognostic performance of the three IR indices for evaluating 360-day mortality in IS patients. TyG-SI demonstrated better predictive capacity compared to the other two parameters. First, TyG-SI maintained robust positive associations with mortality across multiple time horizons, including 360-day, 180-day, and in-hospital periods. Second, ROC curve evaluation revealed TyG-SI’s superiority over TyG and TyG-Lac for all-cause mortality prediction (360-day: 0.605 vs. 0.566 vs. 0.587; 180-day: 0.598 vs. 0.577 vs. 0.586; in-hospital mortality: 0.601 vs. 0.563 vs. 0.578). In addition, significant prognostic relationships were evident when TyG-SI was evaluated either as a continuous measure or a categorical variable. TyG-SI was the only index among the three that significantly enhanced the base model’s predictive performance for both short-term (in-hospital) and long-term (360-day) mortality. TyG-SI may be a more favorable predictor than TyG because the SI may better explain risk factors of insufficient tissue perfusion. Cardiogenic shock can also manifest in normotensive or non-hypotensive patients, although its characteristic manifestations are less pronounced. Compared with non-shocked patients, those with normotensive shock are more likely to exhibit the following features: history of prior ischemic stroke, lower body mass index (BMI), concomitant deep vein thrombosis, reduced left ventricular function, and elevated ventricular systolic pressure [46]. Patients with normotensive shock present with lower SBP and higher heart rate and demonstrate poorer prognosis. The SI provides effective early-phase identification of high-risk ischemic stroke patients, thereby enhancing the predictive value of the individual TyG index. Plasma lactate has traditionally been regarded as a critical biomarker of disease severity and prognosis. In the presence of cardiogenic shock, elevated lactate levels demonstrate robust associations with elevated mortality risk [47,48,49]. For older patients with COVID-19, baseline lactate ≥ 2.0 mmol/L was significantly correlated with ICU mortality, and patients with abnormal lactate levels exhibited higher 30-day and 3-month mortality rates [50]. The composite variable TyG-Lac not only demonstrated favorable performance in predicting all-cause mortality via ROC analysis but also enhanced the predictive accuracy of base risk models for in-hospital mortality. However, recent experimental models and clinical research have indicated that exogenous lactate can improve hemodynamics, attenuate inflammation processes, and enhance organ function in patients with sepsis, brain injury and acute heart failure at the recovery phase [48,51,52]. It suggests that elevated lactate levels do not invariably represent a risk factor. Lactate may exert distinct therapeutic functions at different stages of disease progression, which may potentially complicate the ability of TyG-Lac to achieve superior performance in predicting mortality among IS patients.

In addition, we found that incorporating TyG-SI or TyG into the base prediction model increased the AUC for 360-day mortality (*p* < 0.05), while incorporation of TyG-SI or TyG-Lac enhanced the AUC for in-hospital mortality (*p* < 0.05). These results indicate that TyG serves as a relatively reliable long-term prognostic indicator in IS patients, TyG-Lac demonstrates better predictive value for short-term prognosis, and TyG-SI is applicable for both short- and long-term prognostication.

Although the absolute increase in AUC was modest after adding TyG or TyG-SI to the base model (AUC = 0.701 for the base model, increasing to 0.716 with TyG and 0.723 with TyG-SI) and did not achieve a transformative improvement in discriminative ability, this does not diminish their important clinical value in predicting the prognosis of critically ill ischemic stroke patients. Instead, it highlights their unique advantages and application potential as auxiliary markers. The core value of these indices is not to replace established severity scoring systems (e.g., SOFA, SAPS II), but to serve as simple and efficient supplementary tools to further optimize the accuracy of existing clinical risk assessment models, especially suitable for the clinical management of critically ill ischemic stroke patients.

Early bedside risk stratification of critically ill ischemic stroke patients is faced with the core demand of “rapidity, convenience, and accuracy”. Clinicians often rely on easily accessible physiological and metabolic indicators without additional testing costs to make diagnosis and treatment decisions; TyG and TyG-SI precisely meet this clinical need. Both are entirely derived from routinely measured parameters such as triglycerides, glucose, heart rate, and systolic blood pressure, without increasing additional medical costs or operational burden. They can be quickly integrated into existing risk assessment frameworks to achieve rapid bedside risk stratification and provide timely reference for clinical decision-making. Among them, TyG-SI showed more stable and better prognostic value: it remained independently associated with 360-day mortality in the fully adjusted Cox model, and patient mortality increased gradually with the elevation of its quartiles. Nevertheless, the magnitude of incremental predictive improvement was modest. Moreover, it had the best discriminative ability among the three indices (TyG, TyG-SI, TyG-Lac), indicating its superior specificity and stability in predicting the prognosis of critically ill ischemic stroke patients.

Notably, the clinical significance of TyG and TyG-SI is more reflected in the accurate identification of high-risk patients. The incremental improvement in discriminative ability brought by them can effectively screen out patients who are not fully identified by conventional risk scores and require closer monitoring and intensive supportive treatment, providing an important basis for the formulation of individualized clinical treatment strategies and thus potentially improving the clinical outcomes of these patients. Although the clinical utility cannot be fully quantified by AUC differences alone, the advantages of “convenience, low cost, and accessibility” endow them with irreplaceable application value in clinical practice, combined with the characteristics of clinical management of critically ill ischemic stroke patients. Future studies with external validation cohorts are needed to further verify their value. TyG and TyG-SI are expected to become routine auxiliary markers for bedside prognosis assessment of critically ill ischemic stroke patients, providing support for clinical risk stratification and individualized treatment.

Interestingly, multiple randomized controlled studies, including the SHINE trial, have established that aggressive glucose management in acute ischemic stroke patients, relative to standard therapeutic strategies, does not meaningfully improve 90-day functional outcomes, mortality, or recurrence rates, but instead significantly elevates hypoglycemia risk (OR = 9.46, 95% CI: 4.59–19.50) [53]. This demonstrates that strict glucose regulation by itself is insufficient for improving clinical prognosis in ischemic stroke. Consequently, future clinical trials should focus more on the cardiovascular benefits of novel antidiabetic agents rather than solely on glycemic control. Thiazolidinediones, such as pioglitazone, may reduce vascular complications in IS patients through improvement of insulin resistance [54]. This also suggests that IR indices possess unique value in assessing clinical outcomes of IS, and investigating the effects of novel antidiabetic drugs on IR indices would be of considerable significance.

However, our study had some inherent limitations requiring careful interpretation.

(1) Because of the retrospective observational design, this study can only demonstrate associations and cannot establish causal relationships. Although multivariable adjustment was performed, residual confounding from unmeasured variables remains possible, particularly since important stroke-specific factors —including ischemic stroke subtype, baseline neurologic severity (NIHSS score), time from symptom onset to hospital/ICU admission, acute reperfusion treatment details, and specific causes of death—were unavailable or incomplete in the database. These factors may significantly influence mortality risk and introduce bias. (2) Only the first eligible ICU admission was included, which reduced duplication but may not capture the full clinical trajectory of patients with recurrent admissions. (3) Biomarkers were assessed at a single time point, and serial trajectories were not analyzed. (4) TyG-related indices were derived from early ICU laboratory data, and fasting status could not be confirmed; therefore, these measures are best considered pragmatic surrogate biomarkers in critical care rather than strict fasting-based metabolic indices. (5) Direct measures of insulin resistance, such as HOMA IR, were unavailable. (6) Exclusion of patients with missing data required for TyG, TyG-SI, or TyG-Lac calculation may have introduced selection bias if missingness was not random. Despite the use of multiple imputation for variables with 5–10% missingness, the final cohort may not fully represent the broader critically ill IS population. (7) The study was based on a single critical care database without external validation, which may limit generalizability.

## 5. Conclusions

According to existing research, this is the first study to examine the three IR indices—TyG, TyG-SI, and TyG-Lac for predicting 360-day mortality in critically ill IS patients. In this retrospective cohort of ICU-admitted patients with ischemic stroke, TyG, TyG-SI, and TyG-Lac were associated with 360-day mortality. These findings should be interpreted as associative rather than causal and require confirmation in prospective studies. When incorporated into the base risk model, TyG and TyG-SI improved the prediction of 360-day all-cause mortality, while TyG-SI and TyG-Lac enhanced in-hospital mortality prediction. These findings suggest that TyG-based IR indices represent promising tools for stratifying risk and evaluating prognosis among critically ill IS patients. However, the results should be interpreted cautiously because of the retrospective design, possible residual confounding, and limited generalizability outside the ICU setting.

## Figures and Tables

**Table 1:** Baseline variables of survivors and non-survivors.

Variables	Total (*n* = 812)	Survivor (*n* = 567)	Non-Survivor (*n* = 245)	* p *
Age (years), mean ± SD	63.8 ± 14.0	62.5 ± 14.4	66.8 ± 13.4	0.002 *
Height (cm), mean ± SD	166.7 ± 10.9	166.7 ± 10.1	167.3 ± 5.4	0.732
Weight (kg), median [IQR]	84.1 [67.0, 101.2]	85.3 [67.9, 102.7]	81.3 [66.2, 96.4]	0.135
sex, N (p%)				
0 (female)	357.0 (44.0%)	243.0 (42.9%)	114.0 (46.5%)	
1 (male)	455.0 (56.0%)	324.0 (57.1%)	131.0 (53.5%)	0.333
BMI, median [IQR]	26.8 [22.9, 30.7]	26.3 [21.4, 31.2]	26.7 [22.3, 31.1]	0.521
SOFA, mean ± SD	6.1 ± 3.6	5.7 ± 3.5	7.0 ± 3.9	<0.001 *
APS III, mean ± SD	51.7 ± 22.2	48.7 ± 21.1	58.6 ± 23.3	<0.001 *
SAPS II, mean ± SD	40.7 ± 14.2	38.7 ± 13.7	45.5 ± 14.2	<0.001 *
GCS, mean ± SD	12.7 ± 3.5	12.8 ± 3.3	12.6 ± 3.9	0.468
OASIS, mean ± SD	35.5 ± 8.6	34.6 ± 8.3	37.7 ± 8.9	<0.001 *
Heart failure, N (p%)				0.100
0	560.0 (69.0%)	401.0 (70.7%)	159.0 (64.9%)	
1	252.0 (31.0%)	166.0 (29.3%)	86.0 (35.1%)	
Arterial fibrillation, N (p%)				0.017 *
0	596.0 (73.4%)	430.0 (75.8%)	166.0 (67.8%)	
1	216.0 (26.6%)	137.0 (24.2%)	79.0 (32.2%)	
Respiratory failure, N (p%)				<0.001 *
0	343.0 (42.2%)	279.0 (49.2%)	64.0 (26.1%)	
1	469.0 (57.8%)	288.0 (50.8%)	181.0 (73.9%)	
AKI, N (p%)				<0.001 *
0	416.0 (51.2%)	314.0 (55.4%)	102.0 (41.6%)	
1	396.0 (48.8%)	253.0 (44.6%)	143.0 (58.4%)	
Hemoglobin (g/dL), mean ± SD	11.2 ± 1.9	11.2 ± 2.3	10.9 ± 2.6	0.004 *
Platelet (K/uL), median [IQR]	190 [140, 257]	193 [145, 258]	197 [139, 267]	0.111
RBC (m/uL), mean ± SD	3.8 ± 0.8	3.9 ± 0.8	3.7 ± 0.9	0.010 *
WBC (K/uL), median [IQR]	10.8 [6.8, 17.2]	11.3 [7.9, 16.2]	10.5 [5.9, 18.6]	0.139
Glucose (mg/dL), median [IQR]	146.9 [99.5, 217.1]	142.1 [93.6, 215.9]	158.9 [98.9, 217.7]	0.120
Potassium (mEq/L), mean ± SD	4.3 ± 0.8	4.2 ± 0.8	4.4 ± 0.9	0.018 *
Sodium (mEq/L), mean ± SD	138.9 ± 5.9	139.0 ± 5.9	138.7 ± 5.7	0.583
Calcium (mg/dL), mean ± SD	8.4 ± 0.9	8.4 ± 0.9	8.3 ± 0.8	0.111
Magnesium (mg/dL), mean ± SD	1.9 ± 0.6	1.9 ± 0.2	2.00 ± 0.4	0.469
PT, (s), median [IQR]	14.0 [10.4, 19.0]	13.6 [9.9, 18.5]	15.0 [11.4, 19.7]	0.003 *
PTT, (s), median [IQR]	31.76 [21.0, 44.7]	30.4 [21.1, 42.8]	31.3 [22.1, 45.8]	0.323
Creatinine (mg/dL), median [IQR]	1.17 [0.65, 2.09]	1.10 [0.61, 1.97]	1.24 [0.67, 2.31]	0.087
Urea nitrogen (mg/dL), median [IQR]	22.6 [14.3, 35.8]	21.1 [13.1, 33.8]	26.3 [17.2, 40.1]	0.001 *
ALT (IU/L) median [IQR]	32.2 [11.2, 62.5]	32.8 [11.4, 64.7]	31.4 [11.2, 58.0]	0.421
AST (IU/L), median [IQR]	47.8 [16.3, 90.4]	45.5 [15.5, 94.0]	53.4 [18.3, 85.8]	0.150
Lactate (mmol/L), median [IQR]	1.8 [1.1, 2.8]	1.7 [1.0, 2.6]	2.0 [1.2, 3.2]	<0.001 *
HR (beats/min), mean ± SD	86.5 ± 16.9	85.8 ± 17.1	88.2 ± 16.5	0.060
SBP (mmHg), mean ± SD	123.6 ±19.0	125.1± 19.2	120.1 ± 18.3	<0.001 *
TG (mg/dL), median [IQR]	132.5 [94.8, 185.1]	133.9 [99.1, 180.7]	144.5 [99.1, 198.5]	0.02 *
LDL-C (mg/dL), mean ± SD	96.7 ± 43.2	98.6 ± 44.1	95.4 ± 43.6	0.097
HDL-C (mg/dL), mean ± SD	46.7 ± 22.1	47.3 ± 20.2	45.3 ± 22.4	0.103
LOS in ICU (days), median [IQR]	6.6 [3.6, 12.0]	6.2 [3.3, 11.7]	7.2 [4.1, 12.5]	0.169
LOS in hospital (days), median [IQR]	15.5 [9.0, 26.6]	16.7 [10.0, 28.0]	12.6 [6.8, 23.2]	<0.001 *
TyG, median [IQR]	9.4 [8.8–9.8]	9.1 [8.7–9.7]	9.3 [8.9–9.9]	0.021 *
TyG-Lac, median [IQR]	20.3 [9.4–31.4]	19.10 [9.1–29.1]	23.45 [10.5–36.3]	0.002 *
TyG-SI, median [IQR]	6.76 [5.3–8.1]	6.3 [5.1–7.9]	7.0 [5.8–8.4]	<0.001 *

Statistical significance is indicated as follows: * *p* < 0.05. Continuous variables are presented as mean ± standard deviation (SD) or median (interquartile range, IQR), as appropriate. Comparisons between groups were performed using the Student’s *t*-test or Mann–Whitney U test for continuous variables, and the chi-square test or Fisher’s exact test for categorical variables. Abbreviations: BMI, body mass index; SOFA, Sequential Organ Failure Assessment; APS III, acute physiology score III; SAPS II, simplified acute physiology score II; GCS, Glasgow Coma Scale; OASIS, Oxford Acute Severity of Illness Score; AKI, acute kidney injury; RBC, red blood cell; WBC, white blood cell; PT, prothrombin time; PTT, partial thromboplastin time; ALT, alanine aminotransferase; AST, aspartate aminotransferase; HR, heart rate; SBP, systolic blood pressure; TG, triglycerides; LDL-C, low-density lipoprotein cholesterol; HDL-C, high-density lipoprotein cholesterol; LOS, length of stay; ICU, intensive care unit; TyG, triglyceride–glucose index.

**Table 2:** Association between IR-related indices and 360-day mortality (Cox regression analysis).

Index	Groups	Non-Adjusted HR (95% CI) *p*-Value	Model 1 HR (95% CI) *p*-Value	Model 2 HR (95% CI) *p*-Value
TyG	Continuous	1.68 (1.52–1.76) 0.001 *	1.21 (1.05–1.41) 0.008 *	1.32 (1.05–1.43) 0.007 *
	Q1 (N = 203)	Ref	Ref	Ref
	Q2 (N = 203)	1.25 (0.86–1.82) 0.236	1.29 (0.88–1.89) 0.18	1.34 (0.91–1.99) 0.136
	Q3(N = 203)	1.26 (0.86–1.84) 0.224	1.33 (0.9–1.95) 0.14	1.39 (0.94–2.07) 0.095
	Q4 (N = 203)	1.88 (1.28–2.76) 0.001 *	1.79 (1.24–2.59) 0.002 *	1.64 (1.15–2.36) 0.006 *
	* p * for trend	0.008	0.003	0.002
TyG-SI	Continuous	1.24 (1.05–1.38) <0.001 *	1.14 (1.07–1.21) < 0.001 *	1.11 (1.04–1.19) 0.001 *
	Q1 (N = 203)	Ref	Ref	Ref
	Q2 (N = 203)	0.92 (0.61–1.38) 0.190	0.92 (0.61–1.38) 0.701	0.85 (0.56–1.29) 0.122
	Q3 (N = 203)	1.32 (1.02–1.70) 0.039 *	1.39 (1.06–2.03) 0.08 *	1.25 (1.11–1.84) 0.041 *
	Q4 (N = 203)	1.96 (1.38–2.79) < 0.001 *	2.22 (1.55–3.19) < 0.001 *	1.98 (1.37–2.88) < 0.001 *
	* p * for trend	<0.001	<0.001	<0.001 *
TyG-Lac	Continuous	1.11 (1.08–1.23) < 0.001 *	1.24 (1.07–1.32) < 0.001 *	1.18 (1.01–1.21) 0.003 *
	Q1 (N = 203)	Ref	Ref	Ref
	Q2 (N = 203)	1.31 (0.90–1.91) 0.153	1.30 (0.89–1.91) 0.163	1.30 (0.89–1.91) 0.170
	Q3 (N = 203)	1.17 (0.79–1.73) 0.414	1.18 (1.03–1.40) 0.037 *	1.09 (0.73–1.63) 0.651
	Q4 (N = 203)	1.92 (1.34–2.75) < 0.001 *	1.95 (1.36–2.80) < 0.001 *	1.75 (1.20–2.52) 0.003 *
	* p * for trend	0.023 *	0.011 *	0.008 *

Statistical significance is indicated as follows: * *p* < 0.05. TyG index: Q1 (7.89–8.38), Q2 (8.38–8.97), Q3 (8.97–9.42), Q4 (9.42–11.21). TyG-SI: Q1 (3.61–4.49), Q2 (4.49–5.87), Q3 (5.87–7.07), Q4 (7.07–11.12). TyG-Lac index: Q1 (6.21–9.03), Q2 (9.03–13.36), Q3 (13.36–18.83), Q4 (18.83–68.01). Model 1: Age, arterial fibrillation, RF and AKI. Model 2: Age, arterial fibrillation, RF, AKI, SOFA, APS III, SAPS II, OASIS, hemoglobin, RBC, potassium, prothrombin time, and urea nitrogen.

**Table 3:** Predictive accuracy of IR indices individually and in combination with the base model for mortality.

Models	AUC (95% CI)	* p * Value
360-Day mortality		
TyG index	0.566(0.532–0.592)	ref
TyG-SI	0.605(0.578–0.623)	0.002 *
TyG-Lac index	0.587(0.532–0.614)	0.014 *
Base model	0.701 (0.679–0.724)	ref
+TyG index	0.716 (0.683–0.733)	0.006 *
+TyG-SI	0.723 (0.691–0.748)	0.032 *
+TyG-Lac index	0.708 (0.685–0.712)	0.613
180-Day mortality		
TyG index	0.577(0.553–0.622)	ref
TyG-SI	0.598(0.576–0.631)	0.011 *
TyG-Lac index	0.586(0.567–0.627)	0.020 *
Base model	0.721 (0.683–0.742)	ref
+TyG index	0.707 (0.693–0.733)	0.851
+TyG-SI	0.710 (0.682–0.732)	0.122
+TyG-Lac index	0.709 (0.682–0.726)	0.343
In-hospital mortality		
TyG index	0.563(0.545–0.602)	ref
TyG-SI	0.601(0.576–0.642)	0.004 *
TyG-Lac index	0.578(0.549–0.638)	0.021 *
Base model	0.655 (0.623–0.684)	ref
+TyG index	0.670 (0.654–0.693)	0.483
+TyG-SI	0.687 (0.653–0.703)	0.007 *
+TyG-Lac index	0.661 (0.634–0.685)	0.021 *

Statistical significance is indicated as follows: * *p* < 0.05. The base model included age, arterial fibrillation, respiratory failure, AKI, SOFA, APS III, SAPS II, OASIS, hemoglobin, RBC, potassium, prothrombin time, and urea nitrogen.

## Data Availability

All original data on this article will be provided without reservation, and details can be obtained from the corresponding author.

## Supplementary Materials

The following supporting information can be downloaded at: https://www.mdpi.com/article/10.3390/jcm15072680/s1, Table S1: Proportion of missing data for candidate variables and corresponding handling strategies.

## Abbreviations

IS	ischemic stroke
IR	insulin resistance
RCS	restricted cubic spline
ROC	receiver operating characteristic
TyG	triglyceride–glucose
BMI	body mass index
SOFA	sequential organ failure assessment
APS III	acute physiology score III
SAPS II	simplifed acute physiological score II
GCS	Glasgow Coma Scale
OASIS	oxford acute severity of illness score
AKI	acute kidney injury
RBC	red blood cell
WBC	white blood cell
PTT	Partial Thromboplastin Time
ALT	alanine aminotransferase
AST	aspartate aminotransferase
SBP	Systolic Blood Pressure
TG	triglycerides
LDL-C	low-density lipoprotein cholesterol
HDL-C	high-density lipoprotein cholesterol
