# Supplementary material for: Retrospective Cohort Analysis of TyG, TyG-SI, and TyG-Lac Indices as Predictors of 360-Day Mortality in Critically Ill Ischemic Stroke Patients
Source: J Clin Med. 2026 Apr 1;15(7):2680. doi: 10.3390/jcm15072680 (PMC13073108; doi:10.3390/jcm15072680)
Supplement: Supplementary file 1 [file jcm-15-02680-s001.zip › jcm-4155625-supplementary.pdf]

Table S1. Proportion of missing data for candidate variables and corresponding handling strategies.

Variable	Missing n (%)	Role in analysis	Handling strategy	Included in the fully adjusted Cox model
Age	0 (0.0)	Baseline covariate	Complete data	Yes
Sex	0 (0.0)	Baseline covariate	Complete data	No
Height	28 (3.4)	Baseline descriptor	Median imputation if <5%	No
Weight	21 (2.6)	Baseline descriptor	Median imputation if <5%	No
BMI	24 (3.0)	Baseline descriptor	Derived from height and weight; recalculated when available, otherwise median imputation if <5%	No
SOFA	15 (1.8)	Severity score	Database-derived; imputed if necessary	Yes
APS III	18 (2.2)	Severity score	Database-derived; imputed if necessary	Yes
SAPS II	19 (2.3)	Severity score	Database-derived; imputed if necessary	Yes
GCS	13 (1.6)	Severity score / neurologic assessment	Median imputation if <5%	No
OASIS	17 (2.1)	Severity score	Database-derived; imputed if necessary	Yes

Variable	Missing n (%)	Role in analysis	Handling strategy	Included in the fully adjusted Cox model
Heart failure	7 (0.9)	Comorbidity	Complete-case or mode imputation	No
Arterial fibrillation	7 (0.9)	Comorbidity	Complete-case or mode imputation	Yes
Respiratory failure	8 (1.0)	Comorbidity / clinical condition	Complete-case or mode imputation	Yes
AKI	6 (0.7)	Comorbidity / complication	Complete-case or mode imputation	Yes
Hemoglobin	9 (1.1)	Covariate	Median imputation if <5%	Yes
Platelet count	8 (1.0)	Covariate	Median imputation if <5%	No
RBC	9 (1.1)	Covariate	Median imputation if <5%	Yes
WBC	10 (1.2)	Covariate	Median imputation if <5%	No
Glucose	0 (0.0)	Essential variable for TyG/TyG-SI/TyG-Lac	Patients with missing values excluded during screening	No
Potassium	12 (1.5)	Laboratory covariate	Median imputation if <5%	Yes
Sodium	12 (1.5)	Laboratory covariate	Median imputation if <5%	No
Calcium	14 (1.7)	Laboratory covariate	Median imputation if <5%	No

Variable	Missing n (%)	Role in analysis	Handling strategy	Included in the fully adjusted Cox model
Magnesium	16 (2.0)	Laboratory covariate	Median imputation if <5%	No
PT	18 (2.2)	Laboratory covariate	Median imputation if <5%	Yes
PTT	20 (2.5)	Laboratory covariate	Median imputation if <5%	No
Creatinine	14 (1.7)	Covariate	Median imputation if <5%	No
Urea nitrogen	16 (2.0)	Covariate	Median imputation if <5%	Yes
ALT	26 (3.2)	Laboratory covariate	Median imputation if <5%	No
AST	29 (3.6)	Laboratory covariate	Median imputation if <5%	No
Lactate	0 (0.0)	Essential variable for TyG-Lac	Patients with missing values excluded during screening	No
Heart rate (HR)	0 (0.0)	Essential variable for TyG-SI	Patients with missing values excluded during screening	No
SBP	0 (0.0)	Essential variable for TyG-SI	Patients with missing values excluded during screening	No
TG	0 (0.0)	Essential variable for TyG/TyG-SI/TyG-Lac	Patients with missing values excluded during screening	No

Variable	Missing n (%)	Role in analysis	Handling strategy	Included in the fully adjusted Cox model
LDL-C	31 (3.8)	Lipid covariate	Median imputation if <5%	No
HDL-C	31 (3.8)	Lipid covariate	Median imputation if <5%	No
LOS in ICU	9 (1.1)	Outcome descriptor	Median imputation if <5%	No
LOS in hospital,	8 (1.0)	Outcome descriptor	Median imputation if <5%	No
TyG	0 (0.0)	Main exposure	Derived from complete essential variables in final cohort	Yes*
TyG-Lac	0 (0.0)	Main exposure	Derived from complete essential variables in final cohort	Yes*
TyG-SI	0 (0.0)	Main exposure	Derived from complete essential variables in final cohort	Yes*

TyG, TyG-Lac, and TyG-SI were analyzed as the primary exposure variables in separate fully adjusted Cox models (e.g., Base model+TyG, Base model+TyG-Lac, Base model+TyG-SI) rather than being entered simultaneously into the same model.

Abbreviations: BMI, body mass index; SOFA, Sequential Organ Failure Assessment; APS III, Acute Physiology Score III; SAPS II, Simplified Acute Physiology Score II; GCS, Glasgow Coma Scale; OASIS, Oxford Acute Severity of Illness Score; AKI, acute kidney injury; RBC, red blood cell; WBC, white blood cell; PT, prothrombin time; PTT, partial thromboplastin time; ALT, alanine ami-notransferase; AST, aspartate aminotransferase; HR, heart rate; SBP, systolic blood pressure; TG, triglycerides; LDL-C, low-density lipoprotein cholesterol; HDL-C, high-density lipoprotein choles-terol; LOS, length of stay; ICU, intensive care unit; TyG, triglyceride-glucose index.
